# P-605. Burden of Hospitalizations Due to Respiratory Syncytial Virus in Adults ≥50 Years of Age and Those with Congestive Heart Failure or Chronic Obstructive Pulmonary Disease Exacerbations

**DOI:** 10.1093/ofid/ofaf695.818

**Published:** 2026-01-11

**Authors:** Ashley Tippett, Pragati V Prasad, Sara S Kim, Theda Gibson, Luis W Salazar, Meg Taylor, Olivia Reese, Gabriella Ess, Chris Choi, Caroline R Ciric, Elizabeth Taylor, Khalel De Castro, Samadhan Jadhao, He-Ying Sun, Hui-Mien Hsiao, Shadwal Gupta, Wensheng Li, Kathleen Stephens, Robin Hubler, Qing Liu, Bradford Gessner, Sonal Uppal, Luis Jodar, Caihua Liang, Carol Kao, Nadine Rouphael, Kristin Nelson, Larry Anderson, Chrisina Rostad, Elizabeth Begier, Evan J Anderson

**Affiliations:** Emory University, Atlanta, Georgia; Emory University, Atlanta, Georgia; Emory University, Atlanta, Georgia; Emory University School of Medicine, Atlanta, GA; Emory University, Atlanta, Georgia; Emory University, Atlanta, Georgia; Emory University, Atlanta, Georgia; Emory University, Atlanta, Georgia; Emory University, Atlanta, Georgia; Emory University, Atlanta, Georgia; Emory University, Atlanta, Georgia; Emory University, Atlanta, Georgia; Emory University, Atlanta, Georgia; Emory University, Atlanta, Georgia; Emory University School of Medicine, Atlanta, GA; Emory University, Atlanta, Georgia; Emory University School of Medicine, Atlanta, GA; Emory University, Atlanta, Georgia; Pfizer Inc., Collegeville, Pennsylvania; Pfizer Inc., Collegeville, Pennsylvania; Pfizer, Inc., New York, New York; Pfizer, Champaign, Illinois; Pfizer Vaccines, Collegeville, Pennsylvania; Pfizer Inc, New York, NY; WASHINGTON UNIVERSITY IN ST LOUIS SCHOOL OF MEDICINE, ST LOUIS, Missouri; Emory University School of Medicine, Atlanta, GA; Emory University, Atlanta, Georgia; Emory University, Atlanta, Georgia; Emory University, Atlanta, Georgia; Pfizer Vaccines, Collegeville, Pennsylvania; Moderna, Inc., Cambridge, Massachusetts

## Abstract

**Background:**

RSV is a leading cause of acute respiratory illness (ARI) in older adults and those with comorbidities. Understanding RSV disease burden in these vulnerable populations could guide prevention efforts.

**Table 1:**
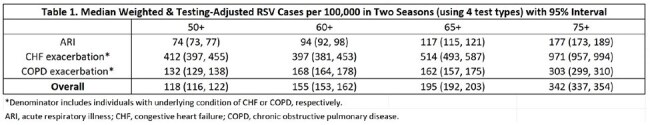
Median weighted and testing-adjusted annual RSV incidence per 100,000 in two respiratory seasons (2018-2019 and 2019-2020) using 4 diagnostic test types with 95% confidence interval.

**Methods:**

We performed active surveillance for RSV among adults aged ≥50 years hospitalized with ARI and adults of any age hospitalized with congestive heart failure (CHF) or chronic obstructive pulmonary disease (COPD) exacerbations within Georgia Health District 3 at two Atlanta hospitals during two respiratory seasons (2018–2019 & 2019–2020). All participants were tested for RSV by BioFire Respiratory Panel (nasopharyngeal & oropharyngeal swabs); subsets provided acute and convalescent sera, cough specimens, and standard-of-care results. Annual population-based incidence of RSV-related hospitalizations was estimated by age strata and study group, adjusting for differences in demographics, diagnostic test results, and test sensitivity. Denominators for ARI and overall incidence estimates were from US Census Bureau. CHF and COPD denominators were limited to those with underlying CHF or COPD, respectively, using NHANES data.

**Results:**

Of 3,090 eligible patients, 1,558 were included in the analysis. Among them, 757 (48.6%) had ARI, 490 (31.4%) CHF exacerbation, and 311 (20.0%) COPD exacerbation. Overall, 92 (5.9%) participants tested RSV positive by any method. Based on data from two seasons, annual population-based incidences (per 100,000 population) among adults aged ≥50 years were 74 (95% CI: 73, 77) for RSV-related ARI hospitalizations only and 59% higher when including all RSV-related hospitalizations (ARI and CHF/COPD exacerbations): 118 (95% CI: 116, 122). Among persons aged ≥50 years with CHF or COPD, respectively, incidences were 412 (95% CI: 397, 455) for RSV-related CHF exacerbation hospitalizations and 132 (95% CI: 129, 138) for RSV-related COPD exacerbation hospitalizations.

**Conclusion:**

RSV was associated with a substantial burden of hospitalizations among adults aged ≥50 years, particularly those with CHF and COPD. Including RSV-related CHF and COPD exacerbation increased overall RSV-hospitalization incidence by >50%. RSV incidence studies should include events beyond ARI or adjustment for not including them.

**Disclosures:**

Pragati V. Prasad, MPH, Moderna, Inc.: Summer Internship Robin Hubler, MS, Pfizer, Inc.: am Pfizer employee and hold Pfizer stocks Qing Liu, M.S., Pfizer Inc.: I am Pfizer employee and hold Pfizer stocks|Pfizer Inc.: Stocks/Bonds (Public Company) Bradford Gessner, MD, Pfizer, Inc.: am Pfizer employee and hold Pfizer stocks Luis Jodar, PhD, Pfizer Inc: Stocks/Bonds (Public Company) Caihua Liang, MD, PhD, Pfizer: I am an employee.|Pfizer: Stocks/Bonds (Public Company)|Pfizer: Stocks/Bonds (Public Company) Nadine Rouphael, MD, Cyanvac: Advisory|Imunon: Advisory|Merck: Grant/Research Support|Moderna: Advisory|Pfizer: Grant/Research Support|Sanofi: Grant/Research Support|Seqirus: Advisory Larry Anderson, MD, AstraZeneca: Advisor/Consultant|Enanta: Advisor/Consultant|GSK: Advisor/Consultant|Janssen: Advisor/Consultant|Pfizer, Inc.: Grant/Research Support Chrisina Rostad, MD, Janssen: Grant/Research Support|Merck: Grant/Research Support|Micron: Grant/Research Support|Moderna, Inc: Grant/Research Support|Pfizer, Inc.: Grant/Research Support|Sanofi: Grant/Research Support Elizabeth Begier, MD, M.P.H., Pfizer: I am an employee.|Pfizer: Stocks/Bonds (Public Company) Evan J. Anderson, MD, Moderna: Stocks/Bonds (Public Company)

